# Catabolic and proinflammatory effects of leptin in chondrocytes are regulated by suppressor of cytokine signaling-3

**DOI:** 10.1186/s13075-016-1112-0

**Published:** 2016-10-03

**Authors:** Anna Koskinen-Kolasa, Katriina Vuolteenaho, Riku Korhonen, Teemu Moilanen, Eeva Moilanen

**Affiliations:** 1 The Immunopharmacology Research Group, University of Tampere School of Medicine and Tampere University Hospital, Tampere, Finland; 2 Coxa Hospital for Joint Replacement, Tampere, Finland

**Keywords:** Leptin, Adipokine, SOCS-3, Osteoarthritis, Chondrocytes, Obesity

## Abstract

**Background:**

Previous studies provide evidence that adipokine leptin increases production of catabolic and proinflammatory factors in chondrocytes and serves as a link between obesity and osteoarthritis (OA). However, the magnitude of the response to leptin treatment varies greatly between chondrocytes from different donor patients. In the present study, we investigated the regulatory role of suppressor of cytokine signaling-3 (SOCS-3) in the leptin-induced responses in OA cartilage.

**Methods:**

Cartilage and synovial fluid samples from 97 patients with OA undergoing knee replacement surgery were collected. Cartilage samples were cultured with leptin (10 μg/ml), and the levels of proinflammatory and catabolic factors in synovial fluid and in the cartilage culture media, and SOCS-3 expression in the cartilage were measured. The role of SOCS-3 in leptin signaling was further studied in H4 murine chondrocytes by downregulating SOCS-3 with siRNA.

**Results:**

Leptin-induced expression of matrix metalloproteinases MMP-1, MMP-3, MMP-13, interleukin-6 (IL-6), inducible nitric oxide synthase (iNOS) and cyclooxygenase-2 (COX-2) were higher in the cartilage samples with low SOCS-3 expression. Accordingly, downregulation of SOCS-3 by siRNA in H4 chondrocytes led to enhanced leptin-induced expression of MMP-3, MMP-13, IL-6 and iNOS. Synovial fluid leptin was associated positively, and cartilage SOCS-3 negatively with synovial fluid levels of MMPs in a multivariate model in obese (body mass index (BMI) >30 kg/m^2^) but not in non-obese (BMI <30 kg/m^2^) patients.

**Conclusions:**

Our results show, for the first time, that SOCS-3 regulates leptin-induced responses in cartilage, and could thus be a future drug target in the treatment or prevention of OA, especially in obese patients.

## Background

Adipokines are cytokine-like hormones produced by adipose tissue and originally discovered to regulate energy metabolism [1, 2]. Their role in inflammation and obesity-related disease, such as type 2 diabetes mellitus and cardiovascular disease, and also in rheumatic disease has attracted increasing interest during the past decade. Leptin was first characterized in 1994 [3] and to date it is probably the most studied adipokine. The circulating levels of leptin are closely associated with the amount of stored body fat and with body mass index (BMI) [4]. Leptin is, however, not only produced by adipose tissue, but also by several other tissues, including cartilage and other joint tissues [5–7]. Interestingly, synovial fluid leptin levels are also correlated with BMI and leptin expression in chondrocytes is increased in obese individuals with OA [5, 6, 8]. The expression of leptin and its functional receptor Ob-Rb is also reported to be increased in chondrocytes in OA, in comparison to healthy chondrocytes [6].

Obesity is a major risk factor for OA [9]. Traditionally obesity has been thought to explain the risk of developing OA due to increased wear-and-tear on weight-bearing joints. However, obesity is also a risk factor for hand OA [10], which points to a systemic factor or factors that mediate the obesity-related impact on cartilage. Leptin, with its strong positive association with body fat stores, fits well in this picture; in fact, increasing evidence supports the role of leptin as a significant factor in the pathogenesis of OA. Leptin has been shown to have direct proinflammatory and catabolic effects on cartilage in experimental settings. We and others have previously shown that leptin enhances production of catabolic enzymes, including matrix metalloproteinase 1 (MMP-1), MMP-2, MMP-3, MMP-9, MMP-13, a disintegrin and metalloproteinase with thrombospondin motifs 4 (ADAMTS-4) and ADAMTS-5 and proinflammatory mediators, such as nitric oxide (NO), interleukin 6 (IL-6), IL-1β, IL-8 and prostaglandin E_2_ (PGE_2_) in chondrocytes, synoviocytes and in cartilage [6, 11–19]. These findings suggest that leptin is not only a bystander of cartilage breakdown, but an active detrimental factor in the pathogenesis of OA.

According to our experience, cartilage from different donor patients respond to leptin treatment in a quite versatile manner: some of the samples produce large amounts of catabolic/proinflammatory mediators like MMPs, IL-6 and NO following leptin treatment, while in some samples leptin-induced changes in the production of these factors are very small. Similar wide variation in the response to leptin is also supported by other studies [17]. A study by Pallu et al. showed that primary chondrocytes received from obese patients with OA respond to smaller amounts of leptin to enhance MMP-13 production than chondrocytes obtained from non-obese patients [17], suggesting that obese individuals might be more susceptible to the harmful effects of leptin on cartilage. However, the mechanisms regulating leptin responsiveness in chondrocytes remain unknown.

Suppressor of cytokine signaling 3 (SOCS-3) belongs to SOCS proteins, which are intracellular molecules that have an important function of limiting excessive inflammatory activation of the innate and adaptive immune system [20]. In inflammatory cells SOCS-3 expression is induced by type I and type II cytokine receptors via the JAK-STAT pathway. SOCS-3 binds to the gp130 subunit of those receptors and inhibits the JAK-STAT pathway, thus forming a negative feedback loop to limit cytokine actions [21]. Interestingly, SOCS-3 is also involved in regulating leptin responsiveness in the central nervous system (CNS) [22].

The metabolic function of leptin is to serve as a sensor of body fat stores for the CNS. Elevation of blood leptin due to calorie intake, whether short-term or long-term, in a lean person normally suppresses food intake, whereas decreased leptin levels due to fasting or loss of adipose tissue lead to increased food intake [23]. In obesity however, elevated leptin does not lead to the expected responses in weight control. This is thought to be due to disturbed leptin signaling, also called leptin resistance. Elevated SOCS-3 expression in the CNS is proposed to be the primary mechanism that causes leptin resistance and subsequent failure in controlling food intake in obesity [22]. Consistently, leptin-deficient mice develop severe obesity [24], whereas SOCS-3 conditional knockout mice are resistant to diet-induced obesity [25]. In humans, genetic leptin deficiency also causes severe obesity, though leptin and leptin-receptor-related mutations are extremely rare [26].

SOCS-3 is also expressed in cartilage [27–29], and we reported previously that its expression is lower in cartilage from obese patients with OA than from non-obese patients [8]. That led us to hypothesize that SOCS-3 could be a significant mechanism behind the variable leptin responsiveness in cartilage samples from different donor patients. We addressed the hypothesis by investigating SOCS-3 expression and leptin responsiveness in cartilage samples obtained from 97 patients with OA. In addition, the role of SOCS-3 expression in leptin signaling was studied by downregulating SOCS-3 by siRNA in chondrocyte cultures.

## Methods

### Cartilage and cell cultures

Cartilage and synovial fluid (SF) samples were collected from 97 patients with OA who were undergoing knee replacement surgery. All patients fulfilled the American College of Rheumatology classification criteria for OA [30]. Cartilage samples were processed for tissue culture as previously described [15]. Cartilage pieces were incubated for 42 hours with or without leptin (10 μg/ml). The concentration of leptin used was chosen based on our previous studies and on existing literature [15, 17–19]. Recombinant human leptin was purchased from R&D Systems Europe Ltd, Abindgon, UK. Synovial fluid (SF) samples from the corresponding patients were also collected at the beginning of the arthroplasty. The SF samples were centrifuged at 4000 g at 4 °C and supernatants were collected and kept at −70 °C until assayed.

The immortalized murine H4 chondrocyte cell line [31], developed in the Laboratory of Experimental Rheumatology, University Medical Center, Nijmegen, The Netherlands, was used in the siRNA experiments. The chondrocytes were cultured at 37 °C in humidified 5 % carbon dioxide atmosphere in Dulbecco’s modified Eagle’s medium (DMEM) with L-glutamine and Ham’s F-12 medium (1:1) supplemented with 5 % fetal bovine serum (all obtained from Lonza Group Ltd, Basel, Switzerland).

### Immunoassays and nitrite measurements

Concentrations of MMP-1, MMP-3, MMP-13 and IL-6 were determined by immunoassays with commercial reagents according to the protocol provided by the manufacturer (human total MMP-1, human total MMP-3, human total MMP-13, mouse total MMP-3 and mouse IL-6 ELISA kits were from R&D Systems; human IL-6 ELISA kit was from Sanquin, Amsterdam, The Netherlands; MMP-1 in SF was determined by Multiplex bead array, Fluorokine® Human MMP Multi Analyte Profiling Base Kit, purchased from R&D systems). Nitrite, stable metabolite of nitric oxide (NO), was measured in the culture media by the Griess reaction [32]. The cartilage culture media samples were filtered through Amicon Ultra 10-K filters (from Millipore, Cork, Ireland) at 14,000 g prior to the Griess analysis in order to remove large proteins that might interfere with the Griess analysis.

### RNA isolation and quantitative reverse transcription/polymerase chain reaction

Culture medium was removed at the indicated time points and total RNA of H4 chondrocytes was extracted with GenElute™ Mammalian Total RNA Miniprep kit (Sigma-Aldrich, St Louis, MO, USA). Total RNA was treated with DNAse (Fermentas UAB, Vilnius, Lithuania) and reverse-transcribed to cDNA using TaqMan Reverse Transcription reagents and random hexamers (Applied Biosystems, Foster City, CA, USA). cDNA obtained from the RT reaction was diluted 1:20 with RNAse-free water and subjected to quantitative PCR using TaqMan Universal PCR Master Mix and the ABI Prism 7000 Sequence detection system (Applied Biosystems). Primers and probes for SOCS-3, glyceraldehyde-3-phosphate dehydrogenase (GAPDH), iNOS, IL-6 and MMP-13 were obtained from Metabion International AG (Martinsried, Germany). The primer and probe sequences and concentrations (Table 1) were optimized according to the manufacturer’s instructions in TaqMan Universal PCR Master Mix Protocol part number 4304449 revision C. The expression of mouse MMP-3 mRNA was measured using TagMan Gene Expression Assay (Mm00440295_m1, Applied Biosystems).

**Table 1:** Primer and probe sequences for quantitative RT-PCR

Gene	Oligunucleotide	Sequence	Conc. (nM)
	Forward primer	GCATGGCCTTCCGTGTTC	300
Mouse GAPDH	Reverse primer	GATGTCATCATACTTGGCAGGTTT	300
	Probe	TCGTGGATCTGACGTGCCGCC	150
	Forward primer	GCGGGCACCTTTCTTATCC	300
Mouse SOCS-3	Reverse primer	AAGCTGCCCCCCTCACA	300
	Probe	CTCGGACCAGCGCCACTTCTTCA	150
	Forward primer	CCTGGTACGGGCATTGCT	300
Mouse iNOS	Reverse primer	GCTCATGCGGCCTCCTT	300
	Probe	CAGCAGCGGCTCCATGACTCCC	150
	Forward primer	TCGGAGGCTTAATTACACATGTTC	900
Mouse IL-6	Reverse primer	CAAGTGCATCATCGTTGTTCATAC	300
	Probe	CAGAATTGCCATTGCACAACTCTTTTCTCA	200
	Forward primer	TTGTGTTTGCAGAGCACTACTTGA	900
Mouse MMP-13	Reverse primer	AACTGTGGAGGTCACTGTAGACTTCTT	900
	Probe	CATCCTGCGACTCTTGCGGGAATC	250

*SOCS-3* suppressor of cytokine signaling-3, *iNOS* inducible nitric oxide synthase, *IL-6* interleukin-6, *MMP-13* matrix metalloproteinase-13, *Conc.* concentration

PCR reaction parameters were as follows: incubation at 50 °C for 2 minutes, incubation at 95 °C for 10 minutes, and thereafter 40 cycles of denaturation at 95 °C for 15 s and annealing and extension at 60 °C for 1 minute. Each experimental reaction was performed in duplicate. The relative mRNA levels of SOCS-3, GAPDH, iNOS, IL-6 and MMP-13 were quantified using the standard curve method as described in Applied Biosystems User Bulletin number 2. To calculate the relative expression of MMP-3 mRNA, the 2^(−ΔΔCT)^ method [33] was used. According to the method, the cycle threshold (C_T_) values for MMP-3 mRNA expression in each sample were normalized to the C_T_ values of GAPDH mRNA in the same sample.

### Western blot

Preparation of cell lysates, SDS-polyacrylamide gel electrophoresis and western blot analysis were carried out as previously described [15]. Mouse monoclonal SOCS-3 antibody (sc-51699), rabbit polyclonal iNOS antibodies (sc-651 and sc-650), goat polyclonal cyclooxygenase-2 (COX-2) antibody (sc-1745) and rabbit polyclonal β-actin antibody (sc-1615R), and secondary horseradish peroxidase (HRP)-conjugated goat anti-mouse (sc-2005), goat anti-rabbit (sc-2004) and donkey anti-goat (sc-2020) antibodies were all from Santa Cruz Biotechnology (Santa Cruz, CA, USA). Rabbit polyclonal MMP-13 antibody (ab39012) was from Abcam (Cambridge, MA, USA). Leptin-induced iNOS and COX-2 expression was determined by running the control and leptin-induced samples side by side and the result is given as fold of change in the β-actin-normalized densitometry value of the leptin-induced versus the control sample.

### Downregulation of SOCS-3 expression by siRNA

H4 murine chondrocytes were seeded at 1 × 10^5^ cells/well in 24-well plates. Cells were incubated for 24 hours and transfected with SOCS-3 siRNA or with non-targeting control siRNA. On-Target SMART pool SOCS-3-specific siRNA (targeting sequences of GGCUAGGAGACUCGCCUUA, GGACCAAGAACCUACGCAU, CUAAUGAAACCUCGCAGAU and GAAGGGAGGCAGAUCAACA) and siGENOME Non-Targeting siRNA were used at 100 nM to transfect the cells using DharmaFECT 1. All transfection reagents were from Thermo Scientific Dharmacon (Lafayette, CO, USA) and transfection was carried out according to the manufacturer’s protocol. The experiments were started 48 hours after the transfection by adding leptin (10 μg/ml) (mouse recombinant leptin from R&D systems) in fresh culture medium.

### Statistical analysis

The chi-square test, unpaired *t* test and Mann–Whitney test (where appropriate) were used to analyze differences between subgroups of the patients. The Wilcoxon test was used to calculate the significance of leptin-induced effects in the cartilage culture.

To analyze the differences in leptin responsiveness in relation to SOCS-3 expression, the samples on each western blot gel were divided to two equal sized groups (low SOCS-3 or high SOCS-3) according to SOCS-3 expression. Median leptin responses, measured as change in the production of MMP-1, MMP-3, MMP-13, IL-6 and NO in the leptin-treated versus control sample, and as fold of change in the expression of iNOS and COX-2, were compared between the low SOCS-3 and the high SOCS-3 groups. Possible intergel differences in SOCS-3 expression were controlled by analysis of variance (ANOVA) in which the leptin response variable (e.g., leptin-induced change in production of MMP-1) was set as a dependent variable, western blot gel (1 to 8) as a grouping variable and SOCS-3 expression as a continuous variable as a covariate. Associations were further tested by adjusting for BMI and age.

Correlation between the factors of interest in SF were determined by Pearson’s correlation analysis. The associations between MMPs or IL-6 and leptin in SF, and SOCS-3 expression in cartilage were further analyzed by ANOVA modeling, by including the variable of interest (SF MMP-1, MMP-3 or IL-6) as a dependent variable, leptin in SF and SOCS-3 expression in the cartilage as covariates and gel number as a grouping factor. The analysis was done in BMI subgroups (obese, BMI >30 kg/m^2^; non-obese, BMI <30 kg/m^2^). Natural logarithms were formed of the leptin response values, SOCS-3 expression levels and SF levels of the measured variables where appropriate in order to have normally distributed variables for the ANOVA modeling and for the correlation analyses.

The data were analyzed by IBM SPSS Statistics 19 (IBM Corporation, NY, USA) and Graph-Pad InStat version 3.00 software (GraphPad Software Inc., San Diego, CA, USA). The results of the siRNA experiments are presented as means (SEM). The statistical significance of these data was calculated by two-way ANOVA with Bonferroni multiple comparisons post-test using Graph-Pad Prism 5 for Windows version 5.04 (GraphPad Software Inc.). Differences were considered statistically significant at *p* < 0.05.

## Results

### Leptin-induced production of proinflammatory and catabolic factors in osteoarthritic cartilage in relation to clinical factors and SOCS-3 expression

**Fig. 1:**
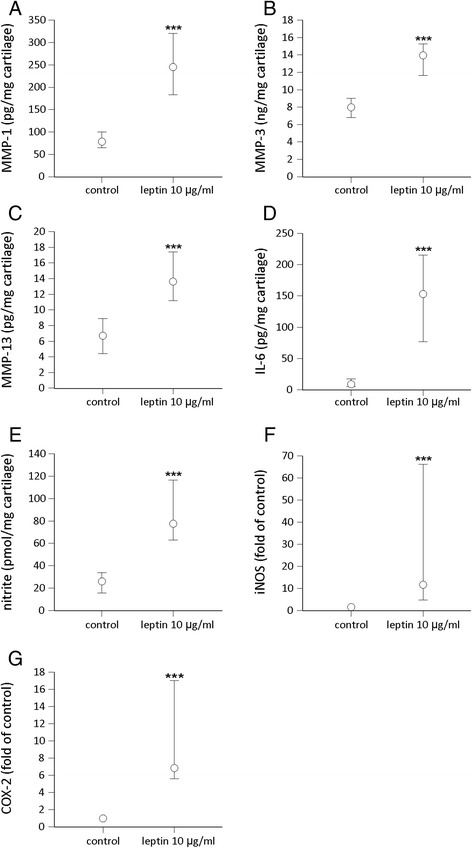
Effect of leptin on the production of matrix metalloproteinase-1 (*MMP-1*) (**a**), MMP-3 (**b**), MMP-13 (**c**), interleukin-6 (*IL-6*) (**d**), nitric oxide (NO) (**e**) and on the expression of inducible nitric oxide synthase (*iNOS*) (**f**) and cyclooxygenase-2 (*COX-2*) (**g**) in cartilage from patients with osteoarthritis (OA). Cartilage samples from 97 patients with OA were cultured with and without leptin (10 μg/ml) for 42 hours. Concentrations of MMP-1, MMP-3, MMP-13 and IL-6 were measured by ELISA; NO production was determined as its metabolite nitrite by the Griess reaction and iNOS and COX-2 proteins by western blotting. The *circles* represent the medians. The *whiskers* represent 95 % confidence interval of the median. Statistical significance was calculated using the Wilcoxon test; ***p < 0.001

Patient characteristics and leptin responses in the cultured cartilage across the whole study population and in the obese (BMI >30 kg/m^2^) and non-obese subgroups are presented in Table 2. Leptin significantly enhanced the expression of MMP-1, MMP-3, MMP-13, IL-6, iNOS and COX-2 and NO production in OA cartilage *ex vivo* (Fig. 1). However, there was considerable variation in these responses between the samples from different donor patients (Table 2). There were no statistically significant differences in the leptin responses between obese and non-obese patients (Table 2), and neither did the leptin responses correlate with age, sex or radiographic scaling of OA.

**Table 2:** Patient characteristics and leptin responses in cartilage cultures in the whole study population and compared across body mass index subgroups

	All		Non-obese, BMI <30 kg/m^2^		Obese, BMI >30 kg/m^2^		
	*n* = 97		*n* = 49		*n* = 48		*P*
Gender (female/male)^a^	60/37		26/23		34/14		0.072
Body mass index (kg/m^2^)^b^	30.9	(6.1)	26.2	(2.4)	35.7	(4.6)	<0.001
Age (years)^b^	69.8	(10.0)	72.8	(9.7)	66.8	(9.4)	0.003
Synovial fluid leptin (ng/ml)^c, d^	12.8	(17.8)	7.6	(11.0)	21.5	(26.7)	<0.001
Synovial fluid IL-6 (pg/ml)^c, d^	118.9	(196.0)	126.8	(204.3)	114.0	(280.2)	0.784
Synovial fluid MMP-1 (ng/ml)^c, d^	14.4	(25.7)	10.4	(16.6)	18.1	(27.3)	0.325
Synovial fluid MMP-3 (ng/ml)^c, d^	649.5	(929.6)	591.6	(571.0)	764.9	(1159.0)	0.106
Leptin response in cartilage							
MMP-1 (change pg/mg cartilage)^c^	145.8	(247.1)	123.2	(253.6)	150.0	(258.3)	0.773
MMP-3 (change ng/mg cartilage)^c^	5.2	(8.8)	6.0	(8.6)	4.9	(10.2)	0.920
MMP-13 (change pg/mg cartilage)^c^	5.8	(13.6)	6.3	(11.8)	5.4	(15.9)	0.983
IL-6 (change pg/mg cartilage)^c^	123.2	(310.2)	114.6	(295.1)	130.9	(312.0)	0.740
NO (change pmol/mg cartilage)^c^	44.5	(133.4)	31.0	(125.8)	52.2	(140.0)	0.359
iNOS (fold of increase)^c, e^	11.7	(160.6)	5.4	(143.1)	15.2	(209.6)	0.501
COX-2 (fold of increase)^c, e^	6.9	(18.4)	7.1	(15.0)	6.4	(22.6)	0.748

^a^Values are numbers of female/male subjects; *p* value was calculated for comparison between non-obese and obese subjects using the chi-square test. ^b^Values are mean (SD); *p* values were calculated for comparison between non-obese and obese subjects using the unpaired *t* test. ^c^Values are median (IQR); *p* values were calculated for comparison between non-obese and obese subjects using the Mann–Whitney test. ^d^Synovial fluid sample was obtained from 90 patients. ^e^Numbers of patients (non-obese/obese) in the analysis were 26/31 for inducible nitric oxide synthase (iNOS) and 25/29 for cyclooxygenase-2 (COX-2). *MMP* matrix metalloproteinase, *IL* interleukin, *NO* nitric oxide

**Fig. 2:**
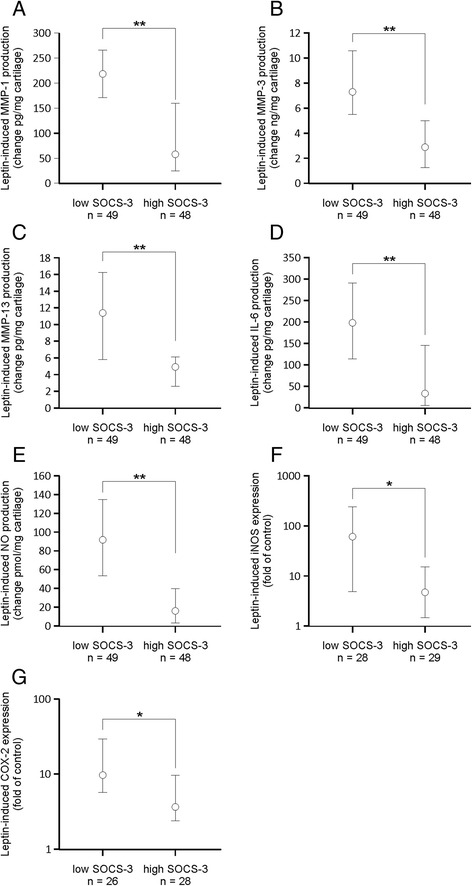
Leptin-induced production/expression of matrix metalloproteinase-1 (*MMP-1*) (**a**), MMP-3 (**b**), MMP-13 (**c**), interleukin-6 (*IL-6*) (**d**), nitric oxide (*NO*) (**e**), inducible nitric oxide synthase (*iNOS*) (**f**) and cyclooxygenase-2 (*COX-2*) (**g**) in cartilage from patients with osteoarthritis (OA) in subgroups stratified by suppressor of cytokine signaling-3 (*SOCS-3*) expression in the non-treated cartilage. Human osteoarthritic cartilage was cultured with leptin (10 μg/ml) for 42 hours. Concentrations of MMP-1, MMP-3, MMP-13 and IL-6 were measured by ELISA, NO was determined as its metabolite nitrite by the Griess reaction and iNOS and COX-2 proteins were analyzed by western blotting. The *circles* represent the median change in the leptin-induced effects. The *whiskers* represent the 95 % confidence interval of the median. Numbers of patients from whom the cartilage samples were collected are indicated. Statistical significance was calculated using the Mann–Whitney test; *p < 0.05, **p < 0.01

When the patients were divided into subgroups according to SOCS-3 expression in the cartilage, leptin-induced changes in the expression/production of MMP-1, MMP-3, MMP-13, IL-6, NO, iNOS and COX-2 in the cartilage were significantly greater in the samples with low SOCS-3 expression than in the samples with high SOCS-3 expression (Fig. 2). This suggests that the level of SOCS-3 expression determinates the magnitude of leptin-induced inflammatory responses. The results remained statistically significant (*p* < 0.05) for the responses in the expression of MMP-3, MMP-13, IL-6, NO, iNOS and COX-2, and almost significant for response in the expression of MMP-1 (*p* = 0.10) in the ANOVA modeling after controlling for intergel variation, BMI and age.

### Synovial fluid levels of MMPs and IL-6 in relation to SF leptin and SOCS-3 expression in cartilage from patients with OA

**Fig. 3:**
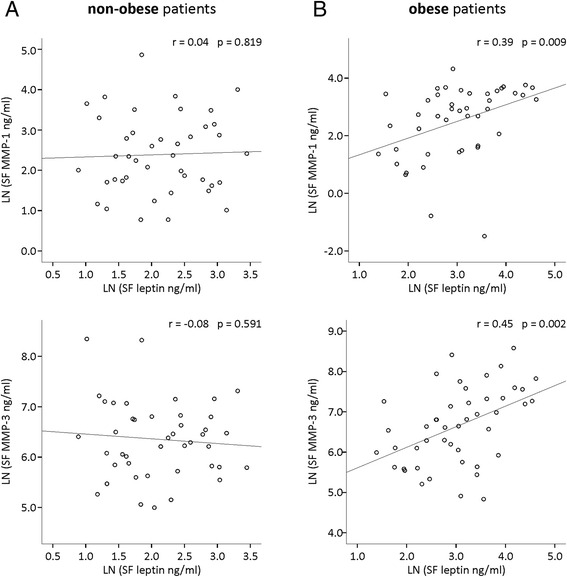
Correlation between leptin and matrix metalloproteinase-1 (*MMP-1*) and MMP-3 in non-obese (**a**) and obese (**b**) patients with osteoarthritis. Leptin and MMPs were measured in synovial fluid (*SF*) by immunoassay. Natural logarithms (*LN*) were formed of the SF levels of leptin and MMPs in order to have normally distributed variables for the Pearson correlation analysis. Correlation coefficients (*r*) and *p* values are indicated. Samples were collected from 90 patients (non-obese, BMI <30 kg/m^2^, *n* = 44; obese, BMI >30 kg/m^2^, *n* = 46)

SF samples were obtained from 90 of the 97 patients. Obese patients had significantly higher SF leptin than non-obese patients, while SF MMP-1 and MMP-3 did not significantly differ between obese and non-obese patients (Table 2). Leptin correlated positively with MMP-1 and with MMP-3 in SF from obese but not from non-obese patients (Fig. 3). In ANOVA modeling, leptin concentrations in SF and SOCS-3 expression in cartilage significantly explained levels of SF MMP-1 and MMP-3 in the obese but not in the non-obese group (Table 3) pointing to obesity-related association of leptin and SOCS-3 in OA pathophysiology. In addition, SF IL-6 levels were explained by SOCS-3 in the obese but not in the non-obese group, while leptin did not significantly explain SF IL-6 levels in either of the BMI subgroups (Table 3).

**Table 3:** Associations between interleukin-6 (IL-6), matrix metalloproteinase-1 (MMP-1), MMP-3 and leptin in synovial fluid and suppressor of cytokine signaling-3 (SOCS-3) expression in cartilage from non-obese and obese patients with osteoarthritis

		Non-obese, BMI <30 kg/m^2^		Obese, BMI >30 kg/m^2^	
Dependent variable	Covariates	*R* ^2^ adjusted	*P*	*R* ^2^ adjusted	*P*
LN (SF MMP-1)		0.15		0.30	
	LN SOCS-3		0.818		0.007
	LN (SF leptin)		0.884		0.023
LN (SF MMP-3)		0.03		0.27	
	LN SOCS-3		0.608		0.004
	LN (SF leptin)		0.733		0.015
LN (SF IL-6)		−0.05		0.20	
	LN SOCS-3		0.945		0.003
	LN (SF leptin)		0.808		0.466

*P* values are calculated for covariates in analysis of variance modeling. The model is controlled for intergel variation in SOCS-3 expression levels. Analysis was performed in body mass index (BMI) subgroups. Natural logarithms (LN) were formed where appropriate. *SF* synovial fluid

### SOCS-3 modulates leptin responses in chondrocytes

**Fig. 4:**
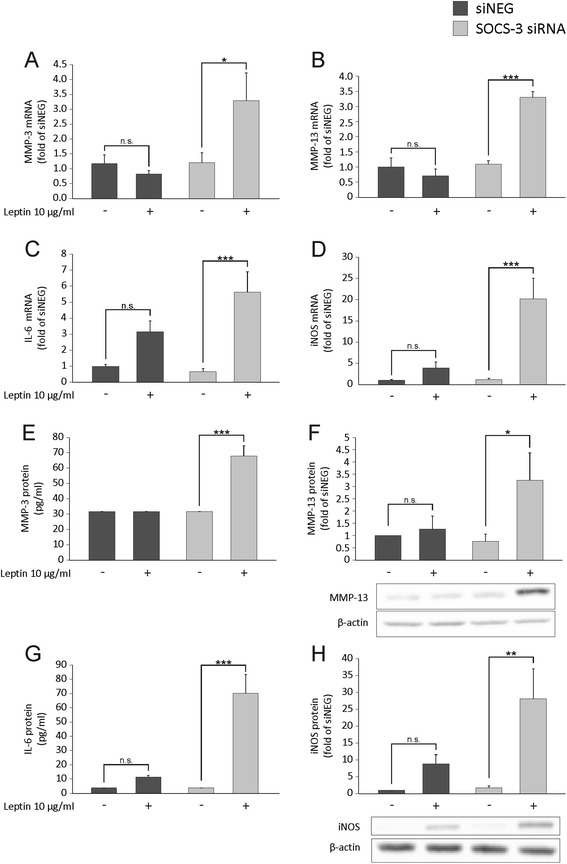
The effect of silencing of suppressor of cytokine signaling-3 (*SOCS-3*) by siRNA on leptin-induced expression of matrix metalloproteinase-3 (*MMP-3*) (**a**, **e**), MMP-13 (**b**, **f**), interleukin-6 (*IL-6*) (**c**, **g**) and inducible nitric oxide synthase (*iNOS*) (**d**, **h**) in H4 murine chondrocytes. The cells were transfected with SOCS-3 siRNA or non-targeting siRNA (*siNEG*) and treated with leptin (10 μg/ml) for 4 (**c**, **d**), 8 (**a**, **b**, **h**) or 24 (**e**-**g**) hours. mRNA expression (**a**-**d**) was determined by quantitative RT-PCR, the levels of MMP-3 (**e**) and IL-6 (**g**) in the culture media supernatants by ELISA, and MMP-13 (**f**) and iNOS (**h**) expression in the chondrocyte lysates by western blotting. Results are expressed as means ± SEM; *n* = 6 (**a**-**e** and **g**) and *n* = 3 (**f**, **h**). MMP-3 protein level in siNEG and in non-treated SOCS-3 siRNA samples was below the detection limit and is set as half of the lowest standard. Representative bands of the western blots are shown. Statistical analysis was carried out by two-way analysis of variance with Bonferroni multiple comparisons post hoc test; *p < 0.05, **p < 0.01, ***p < 0.001. *n.s.* not significant

In order to investigate further the role of SOCS-3 in the regulation of leptin-induced responses in chondrocytes, we used siRNA to downregulate SOCS-3 in the H4 chondrocyte cell line. H4 chondrocytes expressed SOCS-3 mRNA at relatively high levels and it was reduced by approximately 80 % in the SOCS-3-siRNA-treated cells when compared to the cells transfected with control siRNA. Leptin had a clear effect on inducing MMP-3, MMP-13, IL-6 and iNOS expression in the SOCS-3-deficient cells, whereas in the control siRNA-treated cells leptin did not have any statistically significant effect on the production of these factors (Fig. 4), confirming that SOCS-3 negatively regulates leptin-induced proinflammatory responses in chondrocytes.

## Discussion

Leptin has been shown to have detrimental effects on cartilage metabolism in several studies [6, 11–19]. However, considerable variation in leptin responsiveness between cartilage/chondrocytes from different patients has been observed. Our present results indicate that a significant mechanism behind the differential leptin responsiveness could be SOCS-3.

SOCS-3 is a known negative regulator of inflammatory signals [20]. Its role in controlling the effects of leptin in chondrocytes has not been previously investigated, but it has been reported to regulate the responses of leptin in the CNS [22]. In the present study we show, for the first time, that SOCS-3 regulates the proinflammatory and catabolic effects of leptin in chondrocytes. This was demonstrated as greater leptin responsiveness in cartilage explants with low SOCS-3 expression in comparison to lower leptin responsiveness in the explants with high SOCS-3 expression. The causality of this association was illustrated by downregulation of SOCS-3 by siRNA in the chondrocyte cell line, which led to increased leptin-induced expression of proinflammatory and catabolic genes. In addition, SF leptin levels were shown to be positively associated, and cartilage SOCS-3 expression negatively associated with SF MMP levels in obese, but not in non-obese patients with OA. This points to dysregulation of the leptin-SOCS-3 axis, especially in obese individuals, and to a possible obesity-related pathogenic mechanism in OA.

In the present study we observed a positive association between leptin levels and matrix metalloproteinases in SF that was only present in the obese patients with OA. However, obesity did not explain the differential leptin responsiveness in the cartilage culture experiments, unlike in the study by Pallu et al. where greater leptin response in MMP-13 mRNA expression in primary chondrocytes obtained from obese in comparison with non-obese patients with OA was reported [17]. The differences between these two studies could be explained by differential experimental conditions. Instead of primary chondrocytes we used pieces of cartilage, which provides a more natural environment for the chondrocytes in the culture. In addition, we used higher concentrations of leptin to ensure adequate penetration of leptin into the cartilage explant tissue. Of note, in the experimental studies, all cartilage explants were treated with the same leptin concentration in the culture in contrast to the in vivo situation where leptin concentration in SF is highly relative to BMI [5, 6, 8].

Other factors that might affect leptin responsiveness in vivo and that could contribute to the differential leptin responsiveness in cartilage from individual donors as seen in the present study, include functional leptin receptor (Ob-Rb) expression in chondrocytes and the amount of soluble leptin reseptor (sOb-R) in SF or in environment surrounding the chondrocytes. We previously reported that the level of soluble leptin receptor, that is thought to bind active leptin, is decreased in SF in obese subjects [8]. The expression of Ob-Rb has been shown to be unaffected in obesity [6, 17], whereas it has been reported to be increased in severely damaged cartilage [5, 6]. In vivo, all of these mechanisms are likely to contribute to the quantity of leptin-evoked effects, obesity seemingly favoring enhanced responses in many ways.

Leptin binding to its receptor leads to activation of multiple intracellular pathways including the Janus kinase-2 (JAK2)-STAT3, mitogen-activated protein kinase (MAPK), nuclear factor (NF)-kB and PI3K/Akt pathways, all of which have been shown to be involved in the leptin-induced production of proinflammatory factors by chondrocytes [11–15, 18, 19]. SOCS-3 has been shown to inhibit not only the STAT3 pathway, but also the extracellular signal-related kinase (Erk)1/2 and NF-kB pathways [34, 35], providing a possible mechanistic explanation for how leptin-induced responses could be modulated by SOCS-3 in chondrocytes. Interestingly Pallu et al. report higher leptin-induced activation of STAT3 in chondrocytes from obese than from non-obese patients [17], which could be a consequence of decreased SOCS-3 expression, and may explain the differential leptin responsiveness in obese individuals observed in their study. However, the involvement of SOCS-3 expression in explaining increased STAT3 activation in chondrocytes needs to be further studied.

According to the data in the present study, SOCS-3 appears to be a cartilage-protective factor in OA, as low SOCS-3 expression was associated with enhanced proinflammatory and catabolic effects of leptin. Here we investigated the role of SOCS-3 only in leptin signaling, but its role in OA may be much wider, as it can be involved in the regulation of the inflammatory responses induced by a variety of cytokines in multiple cell types in the joint [27, 36]. To our knowledge, only a few groups have previously investigated SOCS-3 expression in chondrocytes. Van de loo et al. reported that SOCS-3 overexpression inhibits lipopolysachharide (LPS)-induced NO production in chondrocytes [29] supporting the idea that SOCS-3 has a similar function in chondrocytes as in white blood cells, that is, to limit excessive inflammatory response. The role of SOCS-3 in arthritis has been studied in animal models by a few groups and the existing data support the idea that SOCS-3 has a protective role in arthritis.

In a study by Shouda et al. *SOCS-3* overexpression by intra-articular adenoviral gene transfer prevented the development of collagen-induced arthritis in mice [37]. Veenbergen et al. reported similar results [38]. In their study *SOCS-3* was delivered into the animals also by adenovirus gene transfer, but intravenously. Conversely, in a study by Wong et al. conditional deletion of *SOCS-3* in the hematopoietic and endothelial cell compartment led to particularly severe arthritis in a mouse model [39], supporting the importance of SOCS-3 as a negative regulator of inflammation. In the first two studies mentioned [37, 38] the target cells of SOCS-3 overexpression were supposed to be synoviocytes, antigen-presenting cells and possibly also B and T lymphocytes. In OA, chondrocytes are thought to be the central cell population that produces pathogenic factors; however, synoviocytes and inflammatory cells are also assumed to contribute to the inflammatory process. The present results indicate that SOCS-3 also modulates the inflammatory response in chondrocytes and thus, could be a promising drug target in the prevention/treatment of OA.

It is unclear, what explains the differential SOCS-3 expression in the cartilage from patients with OA in the present study. Cytokines including IL-1, IL-6, IFN-γ and TNF-α are all known inducers of SOCS-3 [40] and can be also found in the SF of affected joints in OA [41]. Interestingly, anti-inflammatory cytokine IL-10, statins and drugs that elevate cAMP are also known to induce SOCS-3 expression [40]. As it is induced by multiple factors, it is likely that the level of SOCS-3 expression in chondrocytes is defined by a complex net effect of proinflammatory and anti-inflammatory factors. SOCS-3 expression has been reported to be elevated in chondrocytes from patients with OA and RA in comparison to patients with femoral neck fracture without arthritis [29]; this is consistent with previous findings in other tissues, suggesting that SOCS-3 expression is elevated at the sites of inflammation, possibly as a regulatory mechanism to limit excessive inflammation response [42]. It is also possible that SOCS-3 expression would be affected by genetic polymorphisms in *SOCS-3*. There are two single nucleotide polymorphisms (SNPs) previously described in human *SOCS-3*, one in the promoter region and another in the exon 1 of *SOCS-3*. However, in a large case–control study by Hölter et al. [43] these SNPs were found to have no effect on the expression/function of SOCS-3 and there were no differences in the frequency of these SNPs between overweight and underweight individuals.

In the present study leptin was positively associated and SOCS-3 was negatively associated with MMP levels in SF in obese but not in non-obese patients with OA, further confirming the importance of the leptin-SOCS-3 axis in cartilage metabolism and its possible significance in obesity-induced OA. Obesity is a significant risk factor for OA. In a Finnish population-based long-term follow-up study, a sevenfold risk of developing knee OA was reported in obese individuals with BMI >30 kg/m^2^, as compared with subjects with BMI <25 kg/m^2^ [44]. Several epidemiological and cross-sectional studies have investigated the association between leptin and the prevalence/incidence of OA, pain in OA and structural changes in the cartilage in OA [45–55].

Leptin is linked to OA in many of those studies but the results are partly conflicting. The differences might arise from the research frame and also from difficulty in differentiating the impact of body fat stores and leptin by statistical means, as these factors correlate strongly with one another. Karvonen-Gutirrez et al. used sophisticated statistical analysis, by adjusting their data for residuals from the regression of leptin on BMI, aiming to control their data for factors other than the metabolic component of BMI [54]. Interestingly, in their population-based study leptin predicted cartilage defects, as detected by magnetic resonance imaging (MRI) at the 10-year follow-up [54].

Another way to separate the effect of obesity and leptin was attempted by the use of leptin-deficient animals. In fact, Griffin et al. showed that leptin-deficient C57BL mice with diet-induced obesity did not develop OA like the corresponding wild-type mice, suggesting that obesity without increased leptin does not lead to OA [56]. In the present study we analyzed patients with OA in BMI subgroups (obese and non-obese) and interestingly, leptin correlated with MMP enzymes in SF only in the obese patients. This finding further supports the role of leptin in connecting obesity and OA. OA is a heterogenic disease with many known risk factors and it is likely that cartilage destruction in non-obese individuals is driven by pathogenic factors other than leptin. We also showed here that SOCS-3 expression in cartilage is negatively associated with SF levels of IL-6 and MMPs, and this was also seen only in the obese group, suggesting that SOCS-3 expression might be inadequate in the cartilage of obese individuals. Our previous finding that SOCS-3 expression in OA cartilage is decreased in obese compared to non-obese patients [8] supports this idea.

## Conclusions

As a summary of this work, we demonstrated that SOCS-3 negatively modulates the pathogenic effects of leptin in chondrocytes. In addition, SOCS-3 expression in cartilage was negatively associated and synovial fluid leptin levels positively associated with synovial fluid MMP concentrations in obese, but not in non-obese patients with OA. This supports the harmful role of leptin and meaningful regulator role of SOCS-3 in obesity-related OA. Considering these factors in future studies could help to recognize new targets in the treatment and prevention of obesity-related OA.

## Abbreviations

ADAMTS: a disintegrin and metalloproteinase with thrombospondin motifs
ANOVA: analysis of variance
BMI: body mass index
cDNA: complementary DNA
CNS: central nervous system
COX-2: cyclooxygenase-2
C_T_: cycle threshold
DMEM: Dulbecco’s modified Eagle’s medium
ELISA: enzyme-linked immunosorbent assay
Erk1/2: extracellular signal-regulated kinase ½
GAPDH: glyceraldehyde-3-phosphate dehydrogenase
gp130: glycoprotein 130
IFN: interferon
IL: interleukin
iNOS: inducible nitric oxide synthase
JAK: Janus kinase
LN: natural logarithm
LPS: lipopolysaccharide
MAPK: mitogen-activated protein kinase
MMP: matrix metalloproteinase
MRI: magnetic resonance imaging
mRNA: messenger RNA
NF-kB: nuclear factor kappa B
NO: nitric oxide
OA: osteoarthritis
Ob-Rb: functional leptin receptor
PCR: polymerase chain reaction
PGE_2_: prostaglandin E_2_
PI3K: phosphoinositide 3-kinase
SEM: standard error of the mean
SF: synovial fluid
siRNA: small interfering RNA
SNP: single nucleotide polymorphism
sOb-R: soluble leptin receptor
SOCS-3: suppressor of cytokine signaling-3
STAT: a signal transducer and activator of transcription
TNF: tumor necrosis factor
